# Human Macrophage Response to *L. (Viannia) panamensis*: Microarray Evidence for an Early Inflammatory Response

**DOI:** 10.1371/journal.pntd.0001866

**Published:** 2012-10-25

**Authors:** Carolina Ramírez, Yira Díaz-Toro, Jair Tellez, Tiago M. Castilho, Ricardo Rojas, Nicholas A. Ettinger, Irina Tikhonova, Neal D. Alexander, Liliana Valderrama, Janet Hager, Mary E. Wilson, Aiping Lin, Hongyu Zhao, Nancy G. Saravia, Diane McMahon-Pratt

**Affiliations:** 1 Centro Internacional de Entrenamiento e Investigaciones Médicas (CIDEIM), Cali, Colombia; 2 Yale University School of Public Health, New Haven, Connecticut, United States of America; 3 Departments of Internal Medicine and Microbiology, University of Iowa, and the Iowa City VA Medical Center, Iowa City, Iowa, United States of America; 4 Yale University School of Medicine, New Haven, Connecticut, United States of America; Institut Pasteur, France

## Abstract

**Background:**

Previous findings indicate that susceptibility to *Leishmania (Viannia) panamensis* infection of monocyte-derived macrophages from patients and asymptomatically infected individuals were associated with the adaptive immune response and clinical outcome.

**Methodology/Principal Findings:**

To understand the basis for this difference we examined differential gene expression of human monocyte-derived macrophages following exposure to *L. (V.) panamensis*. Gene activation profiles were determined using macrophages from healthy volunteers cultured with or without stationary phase promastigotes of *L. (V.) panamensis*. Significant changes in expression (>1.5-fold change; p<0.05; up- or down-regulated) were identified at 0.5, 4 and 24 hours. mRNA abundance profiles varied over time, with the highest level of activation occurring at earlier time points (0.5 and 4 hrs). In contrast to observations for other *Leishmania* species, most significantly changed mRNAs were up- rather than down-regulated, especially at early time points. Up-regulated transcripts over the first 24 hours belonged to pathways involving eicosanoid metabolism, oxidative stress, activation of PKC through G protein coupled receptors, or mechanism of gene regulation by peroxisome proliferators via PPARα. Additionally, a marked activation of Toll-receptor mediated pathways was observed. Comparison with published microarray data from macrophages infected with *L. (Leishmania) chagasi* indicate differences in the regulation of genes involved in signaling, motility and the immune response.

**Conclusions:**

Results show that the early (0.5 to 24 hours) human monocyte-derived macrophage response to *L. (Viannia) panamensis* is not quiescent, in contrast to published reports examining later response times (48–96 hours). Early macrophage responses are important for the developing cellular response at the site of infection. The kinetics and the mRNA abundance profiles induced by *L. (Viannia) panamensis* illustrate the dynamics of these interactions and the distinct biologic responses to different *Leishmania* species from the outset of infection within their primary host cell.

## Author Summary

*Leishmania* parasites cause a spectrum of diseases (cutaneous, visceral and the deforming forms—chronic cutaneous and mucocutaneous) known as leishmaniasis. The macrophage, a key cell in the immune system, is the cellular target of *Leishmania* parasites in the mammalian host. Previous studies showed the responses of monocytederived macrophages from naturally infected humans to infection with *Leishmania (Viannia) panamensis* were key to adaptive immune responses and clinical outcome. Consequently, an mRNA microarray approach was employed to assess the changes in macrophage gene expression over time (0.5 to 24 hours) induced by *L. panamensis*. The highest level of gene expression induction occurred early (0.5–4 hours); the early pathways (groups of genes) activated included those involved in the innate immune response (signaling, phagocytosis, TLR activation, and inflammatory). Early gene activation is presumed to be important for the developing cellular milieu at the site of infection. By 24 hours post-infection the dominant pathways involved metabolic functions. However, a comparison of the macrophage response to *L. (V.) panamensis* to that of *L. (L.) chagasi* (causative agent of visceral leishmaniasis) at 24 hours revealed a differential up-regulation of genes (cell adhesion, signaling, and inflammation) in response to these species. These observations underscore the distinct biology of different *Leishmania* species from the outset of infection.

## Introduction

*Leishmania* species are obligate intracellular parasites, of extensive public health importance in tropical and subtropical regions of the world. *Leishmania* organisms produce a wide spectrum of diseases.Depending upon the species initiating infection and on the immunological status of the host, disease manifestations range from simple cutaneous to chronic cutaneous, mucocutaneous, diffuse cutaneous and visceralleishmaniasis [3], [4]. Overall control of leishmaniasis has been associated with amoderate type1 CD4 response (IFNγ); whereas excessive down-regulation of this response through the action of various type 2 associated cytokines (e.g. IL-13, IL-10, IL-4) has been related with disease exacerbation.

However, the mechanisms underlying pathogenesis remain to be fully understood.Notably parasites in the *Leishmania (Viannia)* subgenus, which are prevalent in Central and South America and are associated with cutaneous (limited and chronic) as well as mucocutaneous disease, have not been extensively studied. Consequently, the mechanisms involved in pathogenesis are not as yet well characterized. In the case of mucosal disease, a hyperinflammatory response (IFNγ, TNFα) is evident with a down-regulation of IL-10R expression [5]–[8]. In contrast, a mixed cytokine response is observed across the spectrum of cutaneous infection (asymptomatic to chronic) caused by *L. (Viannia)* parasites, both IL-10 and IL-13 have been implicated in disease [1], [7], . Recent studies utilizing a murine model of chronic infection havecorroboratedthe mixed cytokine response and role of IL-13 and IL-10 in the pathogenesis of*L. (V.) panamensis* infection [13].

Differences in parasite gene expression patterns and differences in virulence factors (between various *Leishmania* species and between strains of the same species) undoubtedly contribute to the variation in leishmanial disease manifestations. However, genomic sequences [14] and indeed RNA expression profiles between the promastigote (insect stage) and amastigote (intracellular mammalian stage) have revealed a limited number of differences [15]–[17]. Whereas the relationships between developmentally regulated *Leishmania* genes and parasite virulence or disease associated pathology are of interest, an alternate approach involves the investigation of parasite interactions with host cells. Such studies can provide insight into the underlying mechanisms of the host-parasite dynamic.

Central to the host-*Leishmania* relationship is the macrophage. These cells are fundamental to host defense through their functions in phagocytosis, destruction of the parasites and antigen presentation. *Leishmania* evade the host immune response and replicate inside macrophages establishing a long-term-infection, influencing macrophage gene expression and consequently, the microenvironment in which they persist. The macrophage response to infection is clearly involved in determining susceptibility/resistance in murine leishmaniasis [18]–[22]. The *NRAMP1 or SCL11A1* gene encodes a macrophage transporter [23]–[26], that regulates murine innate susceptibility/resistance to *L.(L.)donovani* and also *L.(L.)mexicana* [26]and has been linked to susceptibility in human visceral leishmaniasis [27]. Further, susceptibility of specific mouse strains to infection parallels the ability of their macrophages to control infection [21], [22], [28]. Studies of *Leishmania*-infected mouse and human macrophages indicate that extensive changes in mRNA abundance occur upon infection [29]–[31] and may be critical to determining the inflammatory and developing host responses.

Individuals with chronic or recurrent human cutaneous disease caused by *Leishmania (Viannia)* have increased *in vitro* susceptibility of monocyte-derived macrophages (MDMs) to parasite entry and survival [1], [32], [33]in comparison to individuals with asymptomatic infection. *In vitro* proliferation of T lymphocytes in response to intracellular amastigotes was found to be significantly lower among individuals with a history of recurrent disease compared with subclinically infected individuals [1]. Furthermore, linear regression analyses generally revealed a relationship between the production of IFNγ, IL-13 and IL-10 by PBMCs of infected individuals [13]; however, this did not occur for cases of recurrent disease [1]. Thus, there are differences in both the innate macrophage and acquired immune responses between individuals with different outcomes of infection.

Previous studies that have examined the response of human monocyte-derived macrophages to *Leishmania* infection have primarily focused on Old World species [2], [15], [29], [34], which lead to distinct immune responses upon infection in comparison to New World species [35]. The gene expression pattern of primary human monocyte-derived macrophages to species of the *Viannia* subgenus has not previously been examined. Herein, we examine the changes in gene expression profiles in response to *Leishmania (Viannia) panamensis* exposure/infection in human MDMs from healthy donors using human gene microarrays. The study indicates a rapid parasite-induced activation of an MDM profile that dampened with time. Pathway analyses indicated an intrinsic activation of signaling and inflammatory responses. In addition, comparisons at 24 hours post-infection to *L.(L.)chagasi* infected human PBMC-derived monocyte-derived macrophages showed thatmolecules involved in cell signaling, adhesion, and inflammation that are evident in *L. (V.) panamensis* infection are generally absent or reduced in the MDM response to *L.(L.) chagasi*. These data suggest the activation/expression profiles of human MDMs differ after interaction with different *Leishmania* species and support the hypothesis that distinct functional activitiesof the host cells may determine the course and outcome of infection with distinct *Leishmania* species.

## Materials and Methods

### Parasite culture

*Leishmania (Viannia) panamensis* strain MHOM/COL/85/1166 was propagated and infective stationary phase promastigotes obtained as previously described [33]. Briefly, promastigotes were cultured in Senekjie's medium at 25°C, recovered on day 6 (stationary phase) by centrifugation at 900× g for 10 min., washed in buffered saline solution (PBS) and used for infection.

### Culture and infection of monocyte derived macrophages (MDM) with *L. (Viannia) panamensis*

All procedures were conducted in accordance with good clinical practice and IRB approved protocols (CIDEIM and YaleUniversity; DMID, NIH). Signed informed consent was obtained according to international guidelines and national regulations. Blood (150 mls) was collected by venipuncture from 6 healthy donors. Donors originated from areas that were non-endemic for leishmaniasis and were non-responsive to leishmanial antigen, as assessed by *in vitro* proliferation [33]. As previously described [33], blood was defibrinated and mononuclear cells were recovered from a Ficoll-Hypaque gradient. Cells were allowed to adhere for 2 hours in flasks coated with gelatin. After washing, adherent cells were differentiated to macrophages for 5 days in culture (37°C, 5% CO_2_) in RPMI 1640 medium supplemented with 10% FBS [33]. Monocyte-derived macrophages (MDMs) were infected at 34°C with *L. (V.) panamensis* promastigotes (opsonized with inactivated AB+ serum) at a 1∶20::MDM∶parasite ratio. Uninfected monocyte-derived macrophages served as controls. Cells were washed at 0.5 hours and the medium replaced. For analysis, cells were harvested at 0.5, 4 and 24 hours and RNA isolated as described below. To assess the level of infection, monocytes from each donor were cultured, differentiated and infected in chamber slides. Infection was microscopically evaluated at each time point, as described previously [1], [33]. Organisms were observed within macrophages by 25 minutes; over the time period of the experiment infection levels did not significantly differ (varied from58–46% of total macrophages) and averaged values of 3.1, 2.8 and 3.2 parasites/macrophage (at 0.5, 4, 24 hours respectively).

### RNA extraction, amplification and cDNA synthesis

At the time points indicated TRIzol (Invitrogen) was directly added to each flask containing the MDMs. RNA was recovered using an RNeasy Micro Kit (Qiagen) and purified according to the manufacturer's specifications. RNA was quantified using a Hitachi GeneSpec III spectrophotometer; quality was assesses using 2 to 3 pg of total RNA and an RNA Pico Chip run on a 2100 Bioanalyzer system (Agilent Technologies). RNA was stored at −70°C until used for microarray analysis.

Polyadenylated RNA was amplified from total RNA using the SenseAMP plus RNA Amplification Kit (Genisphere). Subsequently, cDNA was synthesized from poly (A)+ RNA (1 µg) using an Array 900MPX kit (Genisphere) following manufacturer's recommendations. Reverse transcription was performed for 2 hours at 42°C using SuperScript II (Invitrogen). The reaction was stopped with 0.5 M NaOH/50 mM EDTA; and DNA/RNA hybrids were denatured for 15 min at 65°C. The reaction mix was then neutralized and cDNA was purified using a Qiagen MinElute PCR Purification Kit. Results from three separate comparative microarray analyses (data not shown), indicated that data from amplified and non-amplified RNA samples were comparable; consequently, amplified RNA was used for analysis.

### Microarray hybridization

cDNA was ligated to either Cy3 or Cy5 dye; Cy3 was used for control samples (uninfected monocyte-derived macrophages at identical incubation time) and Cy5 for *L. (V.) panamensis* infected monocyte-derived macrophages. Each tagged (Cy3 or Cy5) cDNA was purified using Qiagen-MinElute PCR Purification Kit. After addition of 1 µg of denatured human Cot-1 DNA to the purified tagged cDNA, the mixture was hybridized to a 70-mer OHU28K Human oligo array (Yale University Keck Microarray Resource)using a 22×71 mm AdvaCard overnight at 55°C using an Advalytix Array Booster Autohyb Instrument. The OHU28K Human Oligonucleotide array was fabricated from a 70 mer oligonucleotide set consisting of 21,329 oligonucleotides representing Operon Human Version 2.0 set plus a Version 2.1 Upgrade set of 6,228 oligonucleotides that represent probes from Version 3.0 Human Genome Set. After hybridization, arrays were washed with 2X SSC buffer and/or 0.2% SDS. Arrays were then dried and incubated for 4 hours with theGenisphere3DNA Array 900MPX capture reagent mix at 65°C. After washingusing 2X SSC buffer containing 2% SDS, arrays were dried and analyzed, as indicated below.

### Gene expression data analysis

Scanning of each array was performed according to manufacturer's instructions using the Axon GenePix 4000A laser confocal scanner at 532 and 635 nm. Genisphere dendrimer cy3/cy5.tiff images and .gal files were analyzed using GenePix Pro Software to grid the images and generate a spreadsheet data.gpr file.

GeneSpring GX 7.2 (Agilent Technologies, Inc., Palo Alto, CA) was used for gene expression data analysis. LOWESS normalization was performed [36] to eliminate dye-related artifacts. Gene expression comparisons were made between infected and uninfected at different time points. In each comparison, genes with the average intensity values less than twice background intensity, or fold change less than 1.5 fold (up or down-regulated) were excluded in the further statistical analysis. A two-sample *t* test using p-value cutoff 0.05 was applied to determine if a gene was statistically differentially expressed. Genes were annotated with KARMA (Yale University, http://medicine.yale.edu/keck/ymd/downloads.aspx) and classified into different GO SLIM categories. Pathway analysis was performed to identify significantly affected pathways using the pathway database from the Yale Center for Statistical Genomics and Proteomics, http://bioinformatics.med.yale.edu/), which includes pathways from KEGG (http://www.genome.jp/kegg/pathway.html), GenMAPP(http://www.genmapp.org/) and Biocarta(http://www.biocarta.com/genes/index.asp) pathway databases. The cutoff to determine genes that were differentially expressed or to cluster similar expressed genes in significant pathways were 1.5- fold difference between the fluorescence signal intensity from a non-infected control and the *L*.(*Viannia*) *panamensis*-infected monocyte-derived macrophage with a significance level of ≤0.05. Although amongst these pathways there are genes in common (such as NF-κβ, JUN, FOS, MYC, PLCB1), the pathways are defined by distinct subsets of genes.The raw data and processed data have been made available at Gene Expression Omnibus (GEO/NCBI) website (GEO ACCESSION NUMBER GSE25819).

For comparative analyses of the monocyte-derived macrophage responses, *L. (L.) chagasi/infantum* (16 hours after infection) was compared to the 24 hour response to *L. (V.) panamensis*. Macrophages in both cases were derived from human peripheral blood monocyte-derived macrophages, using standard methods and infection ratios were comparable. For comparative analyses the microarray fluorescence values from the study of Ettinger and Wilson using Affymetrix U133Plus2 microarray chips [2], adjusted for background intensities (normalized) using the gcRMA method, were employed. For each probe set, the fold change (infected/uninfected) was calculated for each individual in the study, and their geometric mean taken. The data were linked to the 24-hour dataset of the current study by Gene ID (LocusLink). Where more than one probe set or oligonucleotide was present for a single Gene ID, the most extreme of the values was taken. In comparisons with *L*. (*L.*) *chagasi*, genes were considered if, in either study, the multiplicity-adjusted p-value was less than 0.05 for comparing infected versus uninfected. The current study used the Benjamini and Hochberg adjustment [37]. This analysis was done using the R software (http://www.R-project.org). The 24 hour time point was employed, as given the variation in macrophage gene expression found with time post-exposure, would represent comparable experimental conditions. It should be noted that given the decrease in gene expression levels with time([30], this study), the number of genes with transcripts up-regulated would be lower for *L. V. panamensis* at 24 hours (in comparison to 16 hours*(L. chagasi/infantum)*).

### Quantitative PCR validation of the differential gene expression

RNA and cDNA prepared from donors for the microarray analyses was not available in sufficient quantity to conduct the qPCR validation. Therefore MDMs were derived from blood monocytes of 6 to 7 additional normal donors for the validation analyses. cDNA was generated from RNA obtained from MDMs infected with *L.(V.) panamensis* and used as template for the RT-qPCR in an ABI PRISM 7000 Sequence Detection System (Applied Biosystems, Foster City, CA). The reactions were performed using iQ SYBR Green Supermix (Bio-Rad Laboratories) with oligonucleotides at 100 nM. The RT-qPCR settings included an initial activation of DNA polymerase at 95°C for 10 minutes, followed by 50 cycles of denaturation at 95°C for 15 seconds and assay-specific annealing temperature and extension times (Table S1). These SYBR-green assays were used to determine the relative abundance of the following genes: interleukin-1 β (IL-1β), tumor necrosis factor α (TNFα), colony-stimulating factor 2 (granulocyte macrophage) CSF2/GMCSF), prostaglandin-endoperoxide synthase 2 (prostaglandin G/H synthase and cyclooxygenase – PTGS2), and interleukin 6 (IL-6). Following Vandesompele *et al.* [36], the gene expression was normalized by the geometric mean of two internal control genes selected from the microarray analysis that displayed no variation in gene expression, namely, glyceraldehyde-3-phosphate dehydrogenase (GAPDH) and the39S ribosomal protein L18 (RL18), mitochondrial precursor. Each amplicon was cloned in PCR2.1-TOPO (Invitrogen, Carlsbad, CA) with the TOPO TA Cloning Kit (Invitrogen); the generated plasmids were used to generate a standard curve to determine the absolute number of transcripts for each gene in the samples. The number of transcripts for each gene was then normalized to the geometric mean of number of transcripts of GAPDH and RL18. Following the same analyses method applied to the microarray data, for each gene the normalized number of transcripts in the infected MDMs was divided by the normalized number of transcripts in the non-infected MDMs and the geometric mean was calculated for the ratios obtained for the samples from different donors.

## Results and Discussion

### Time course and response to infection/exposure

The infection of macrophages with a parasitic protozoan, such as *Leishmania*, is a complex event involving multiple cellular processes (including phagocytosisand the induction of cell signaling), that result in the expression of cytokines, and chemokines, and that ultimately impact upon the induction of the host immunological response (adaptive immunity). Furthermore, monocyte-derived macrophage physiological and metabolic responses change in response to the parasitism. In order to examine the development of the initial *L. (Viannia) panamensis* promastigote – monocyte-derived macrophage interaction, we studied the changes in gene transcripts at 0.5, 4 and 24 hours of infection/exposure using monocyte-derived macrophages from six healthy non-immune donors. Confounding adaptive immune response by residual lymphocytes was obviated by choosing donors known to live in areas non-endemic for leishmaniasis who were determined to be non-responsive to leishmanial antigen.

**Figure 1:**
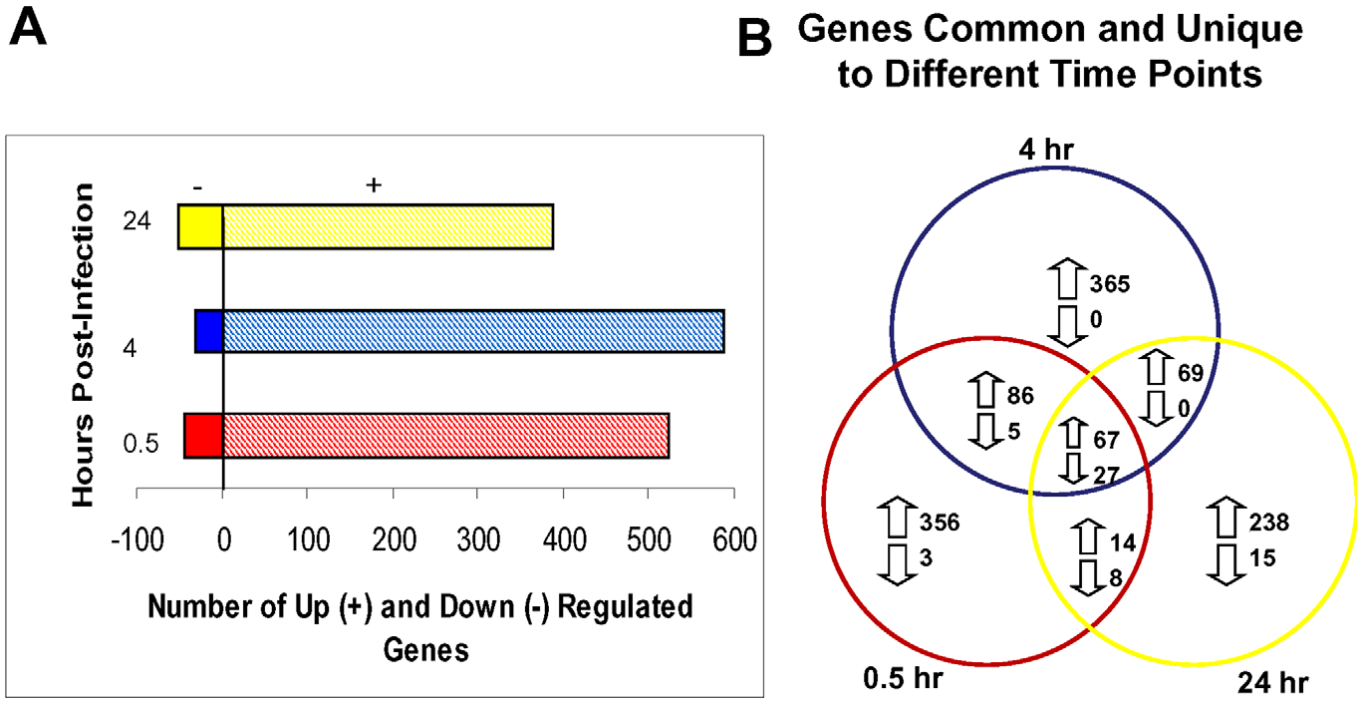
Variation of up- and down-regulated transcripts with time post-infection. A) The number of gene transcripts up- or down-regulated at 0.5, 4 and 24 hours post-infection/interaction with *Leishmania (Viannia) panamensis* promastigotes are schematically summarized. The decreasing response of the up-regulated gene transcripts with time is evident; the number of down-regulated genes is relatively consistent across the time points. B) The numbers of gene transcripts in common or differentially expressed at each time point. Gene transcript expression comparisons were made between infected and uninfected macrophages for each individual at the different time points. Gene transcripts with average intensity values less than 2× background intensity for both comparison groups, or fold change less than 1.5 fold (up or down-regulated) were excluded in further statistical analysis. A paired *t* test using p-value cutoff 0.05 was applied to determine if a gene was statistically differentially expressed.

MDM transcripts that were significantly up- or down-regulated as a consequence of interaction with *L. (Viannia) panamensis* (Figure 1) included more than 500 genes. This response was attenuated at later time points. Notably, at the earliest observational points (0.5 and 4 hours of infection/exposure), a total of 523 and 587 genes, respectively, were found to be up-regulated, whereas by 24 hours this number had declined to 388. The geometric mean of fold-change in expression of the up-regulated transcripts also decreased with time going from 4.39 (0.5 hours) to 3.21 (4 hours) and 2.53 (24 hours). The number of transcripts that were significantly down-regulated remained relatively constant in response to infection/exposure with 43, 32 or 50 transcripts down-regulated at 0.5, 4 or 24 hours, respectively. In addition, the overall geometric mean-fold magnitude of transcript down-regulation was similar overtime (0.0636 (0.5 hours), 0.0366 (4 hours), 0.0486 (24 hours)). Whereas 26 gene transcripts were consistently down-regulated, the expressions of 55 transcripts were observed up-regulated at all three time points. Many of the consistently up- or down-regulated mRNAs encoded proteins involved in cell signaling or gene regulation (**Table S2**).

Consistent with the apparent dampening of the response to infection between 4 and 24 hours after infection, the number of highly up-or down-regulated transcripts (expression>5-fold) decreased with time. At 0.5 hours post-infection/exposure, 210 transcripts were found to be highly (≥5-fold) changed, whereas at 4 or 24 hours of infection the number of highly changed transcripts decreased to 151or 104, respectively. It was notable that amongst the highly regulated transcripts, many were changed at only one time point, with 105, 49 and 23 genes being unique to the 0.5, 4 and 24 hour time points, respectively, underscoring the changing dynamic of the macrophage response.

Although fewer transcripts (**Figure 1**) were down-regulated than up-regulated, a higher proportion of the down-regulated transcripts were consistently down-regulated (52 to 81%, dependent on the time point).Genes encoding these transcripts (**Table S2**)primarily encoded molecules involved in regulation of cell signaling (protein kinases, phosphatases) or molecular transport. Additionally, some of the genes whose expression was down-regulated in *L. (Viannia) panamensis* infected MDMs encoded proteins potentially affectingthe host's immunological control of the invading parasite. These include the receptor for MCP-1 (a known activator of macrophage mediated leishmanial killing [37]–[39]) and the extracellular matrix protein, spondin 2, which has been shown to be involved in the initiation of the innate immune response [40], [41].

During the first 24 hours of infection, the majority of changes were transient; the gene expression profiles reflected a changing dynamic of the host-parasite interaction over the first 24 hours of infection. Whether these differences are due to parasite molecules derived from rapidly killed/damaged parasites or living *Leishmania* remains to be firmly established.The patterns of gene regulation were further examined using pathway analysis (below).

### RT-PCR validation

In order to validate the microarray data, the expression of selected genes was also evaluated using quantitative RT-PCR (**Table 1**). The genes selected for examination (0.5 and 4 hours post-infection/exposure) were chosen because of their potential pertinence to the early macrophage innate response and/or development of the adaptive immune response. These genes are: interleukin-1β (IL-1 β), tumor necrosis factor-α (TNFα), granulocyte-macrophage colony-stimulating factor (GMCSF), prostaglandin-endoperoxide synthase 2 (prostaglandin G/H synthase and cyclooxygenase; PTGS2), and interleukin 6 (IL-6) (**Table S2**).

**Table 1:** Validation of transcript expression of *L. (V.) panamensis*-infected human monocyte-derived macrophages.

GENE	Real Time PCR Analysis		Microarray Data Analysis	
	30 min	4 hrs	30 min	4 hrs
IL1-β	1.74(0.65–4.64)	2.33(1.03–5.30)	1.31(0.45–3.83)	11.67(4.38–31.10)
TNF-α	2.00(0.54–7.48)	4.56(1.44–14.41)	1.7(0.96–3.01)	12.09(3.81–38.41)
CSF2(GM)	1.19(0.31–4.63)	3.45(0.62–19.23)	0.7(0.28–1.94)	7.23(0.95–55.10)
PTGS2	3.17(0.88–11.48)	1.94(0.93–4.03)	2.95(1.10–7.91)	16.91(3.40–84.15)
IL-6	1.54(0.39–6.06)	2.40(0.46–12.55)	2.2(0.99–4.85)	5.17(1.66–16.09)

Comparison of transcript expression in human macrophages infected with *L. (Viannia) panamensis* by quantitative real time PCR versus microarray analyses. Relative expression is expressed as a ratio of transcripts in infected cells over uninfected cells at 30 minutes or 4 hours after infection/interaction was determined. The values presented for the quantitative reverse transcriptase real time PCR analyses are the geometric mean of the ratios of macrophage transcripts from 6 to 7 different donors. The gene expression at each time point was normalized by the geometric mean of GAPDH and 39 S ribosomal protein L18 [36]. The genes listed are: IL1-β – interleukin-1β, TNFα – tumor necrosis factor-α, GMCSF2 –colony-stimulating factor 2 (granulocyte-macrophage), PTGS2 – prostaglandin-endoperoxide synthase 2, and IL6 – interleukin 6. The 95%CI are indicated between brackets.

The increase in expression between 30 minutes and 4 hours detected by microarray was corroborated by qRT-PCR for 4/5 of the genes. Although the fold changes in gene expression were more pronounced for some transcripts in the microarray (i.e. IL1-β, PTGS2), this variation is likely attributable to either the differences between hybridization versus qRT-PCR based amplification methods or by the fact that the experimental samples used for qRT-PCR were generated using PBMCs from individuals different than those employed for the microarray analyses [42]–[44]. Notably, the range of the fold-increases (95% confidence levels) found was consistent for the two methods.

In most cases the direction of change at the different time intervals was the same by both methods, although the expression of PTGS2 was up-regulated maximally at the 0.5 hour time point by qRT-PCR, whereas the transcript level was higher at the 4.0 hour time point according to the microarray analyses. The reason for this discrepancy is not clear. The involvement of prostaglandin synthesis is consistent with other studies of macrophage infection/exposure with *Leishmania* parasites [45], [46] as well as our observations of the engagement of the Peroxisome Proliferators via PPARα (0.5, 4 and 24 hours) and Prostaglandin Synthesis and Regulation (GenMapp) pathways (4 and 24 hours)(**Table S3**). Overall, the qRT-PCR analyses corroborate the results from the microarray studies. The mechanisms involved (induction of non-coding miRNAs, regulation of RNA binding proteins) in conferring mRNA stability/instability remain unclear but are of obvious interest for future research.

### Pathway analysis

To determine the biological processes at play, pathway analyses examining the co-ordinate regulation of specific gene groups were performed for each time point. The genes activated and the correlative pathways illustrate the dynamic nature of the *L. (V.) panamensis*-MDM interaction **(Table S3**).The outcome of pathway analysis at the three time points post-infection/interaction is consistent with sequential responses after phagocytosis. Thus, the pathways coordinately regulated are more similar at the 0.5 hour and 4.0 hours time points than at the 24 hour time point. The pathways induced early in infection/interaction participate in the induction of signal transduction due in part to phagocytosis. It should be noted that all pathways indicated were up-regulated, with respect to the transcripts analyzed.

#### Pathways consistently up-regulated in response to *L. (V.) panamensis* infection/exposure

Certain biological pathways were consistently up-regulated throughout the first 24 hours of infection/interaction. Those pathways continuously engaged include: 1) eicosanoid metabolism, 2) oxidative stress, 3) cadmium induces DNA synthesis and proliferation in macrophages, 4) activation of PKC through G protein coupled receptor and 5) mechanisms of gene regulation by peroxisome proliferators via PPARα.

The oxidative stress response pathway elements elicited in response to infection/interaction primarily involved genes participating in the anti-oxidant response rather than the simultaneous up-regulation of genes involved in the repression of reactive oxygen species (ROS) production. Interestingly at 24 hours, the mRNA products of SOD genes were increased 1.65 to 2.4 fold, at the same time as the transcripts encoding monoamine oxidase (MAO-A; 2.3fold), consistent with the preferential production of hydrogen peroxide (H_2_O_2_). Notably, H_2_O_2_ itself has been shown to regulate the transcription factor function of nuclear factor I (NFI) and hence, cytochrome P450 1A1(CYP1A1) activity [47], [48], leading to the repression of ROS producing systems.The apparent activation of the oxidative stress response could indicate that monocyte-derived macrophages, in reaction to infection are protecting themselves against secondary effects of ROS generated to control the parasite. Such findings could lead to speculations as to whether this response enhances the survival of the macrophage or the parasite or both.

Enhanced expression of transcripts of genes involved ineicosanoid metabolism could lead to an increased production of prostaglandins as well as the up-regulation of receptors for these mediators (prostaglandin G/H synthase and cyclooxygenase, arachidonate 5-lipoxygenase-activating protein, prostaglandin E receptor1, thromboxane A2 receptor). Although prostaglandins have known proinflammatory effects, prostaglandins are also known to induce the production of IL-10 [49], [50]. Overall, evidence suggests that the activation of this pathway, as observed for other *Leishmania* species, could lead todampening of the host defense to infection [51]–[57].

The significance of the G-receptor activation pathway up-regulation likely relates to processes involved in phagocytosis [58] and receptor-mediated signaling (NF-kB, protein kinase C, phospholipdase c and calcium-mediated) intrinsic to infection/uptake that have been reported to be induced by phagocytosis of non-infectious particles [58], [59]. PPARα gene induction is associated with fatty acid metabolism and involves the up-regulation of ApoA and lipoprotein lipase, as well as LXRα and cJUN. PPARα expression can lead to the suppression of inflammatory mediators and potentially the development of alternately activated macrophages [60]–[63]. Such changes would be beneficial to the parasite. PPARα is known to up-regulate matrix metalloproteinases (MMPs) [61], [63]; the early up-regulation of transcripts along this pathway (0.5 hours) is therefore consistent with the increased levels of transcripts encoding matrix metalloproteinases (MMP1,2, or 9) observed at 4 and 24 hours post-infection.

#### Pathways up-regulated early after interaction with *L. (V.) panamensis*

Up-regulated expression of transcripts encoding toll-like receptor (TLR) pathway proteins was evident at 0.5 hours andat 4 hours post-infection/interaction (p<10^−6^). TLRs have been implicated in the infections of other *Leishmania* species (*L. major, L. amazonensis*, and *L. donovani*) [28], [64]–[67].

TLR receptors are important elements of the innate response and can be key to the developing adaptive immune response. The leishmanial surface lipophsophoglycan (LPG) has been shown to interact with TLR2 [65]. However, *L. (Viannia)* parasites express low levels of LPG (10–20-fold less than other species of *Leishmania* [68], [69]). Further, our recent studies [70] indicate that *L. (V.) panamensis* elicits a response through TLR4 and intracellular TLR, which leads to the production of cytokines (such as TNFα) as early as 4 hours post-infection. It is possible that other TLRs known to be activated by other species of *Leishmania*, such as TLR9 [66], [67], may also be involved in this response. As the P-8 proteoglycolipid complex that has been found to activate TLR4 [71] is not expressed by *L. (Viannia) spp.* parasites (McMahon-Pratt, unpublished observations), the ligand responsible for TLR4 activation remains to be determined. Interestingly, transcripts associated with the NFkB activation are up-regulated at the 0.5 and 4 hour time points and NF-κB activation has been found to up-regulate TLR2 expression. Increased expression of TLR2 has in fact been observed in human macrophages upon infection with *L. (V.) panamensis* [70]. Hence TLR modulation represents a potential mode of either therapeutic or preventative intervention [72]–[76] to modulate leishmaniasis. A further understanding of these parasite-host interactions is important for the development of such approaches.

Pathway analyses suggested that there are pronounced changes in expression of proteins relevant to signal transduction and macrophage activation in response to phagocytosis and infection by *L. (V.) panamensis*. Up-regulated expression of MAP kinases and NF-kB suggested that these pathways might be activated and similar increases in transcripts encoding several immunological mediators such as IL-6 and TNFα were consistent with this possibility. Increased expression of NF-kB, TNFα and IL-6 are also consistent with the TLR engagement [70]. Interestingly, TNF has also been reported to be selectively induced in the response of murine dendritic cells [77] to *Leishmania (V.) braziliensis*. Both IL-6 and TNFα have been implicated in the pathology associated with human infection by members of the *L. (Viannia)* subgenus [7], [78]–[81]. Genetic studies have revealed a relationship between alleles/SNPs promoting higher levels of IL-6 or polymorphisms near the TNF promoter and disease [81], [82]. These results suggest that the induction of these key cytokines is an early innate response in humans and may be determinate to disease development.

#### Pathways up-regulated 24 hrs after infection by *L. (Viannia) panamensis*

Although the up-regulation of transcripts along some pathways was still evident, by 24 hours post-infection the magnitude of changes observed in many transcripts had diminished overall. The decreased response is consistent with what has been reported for the responses of macrophages at later times (24 to 96 hours) post-infection with other species of *Leishmania* [2], [29]–[31], [46]. However, and in contrast to previous reports at 24 hours post-infection, many transcripts encoding genes related to the host immune response were still up-regulated in response to *L. (Viannia) panamensis* (CCL24 (eotaxin), TRAIL R1, IL3R, integrin β8, integrin β3(CD61), CD109), as well as cellular signaling (GTP binding protein, inositol 1,4,5-trisphosphate 3-kinase C, vasodilator-stimulated phosphoprotein (VASP),Tec protein tyrosine kinase, phospholipase D2, mitogen-activated protein kinase kinase kinase kinase 3 (MAPK/ERK kinase kinase kinase 3), N-myristoyltransferase 2). Furthermore, by 24 hours post-infection, increased expression of genes involved in some metabolic pathways had become evident (**Table S3**). The nature of these metabolic pathways suggested changes in the cellular requirement for energy metabolism, nucleic acid synthesis and anti-oxidants (glutathione pathway). These changes are consistent with macrophage adaptation for the increased metabolic requirements in cells supporting the maintenance and replication of the intracellular *Leishmania*.

Overall, the variation in gene regulation with time is critical in evaluating the macrophage responses to *Leishmania* infection/interaction, as the initial innate immune responses are important for determining the inflammatory and ultimately acquired immune responses. Generally however, only one time point (generally ≥16 hours post-infection; well after these responses have diminished) has been examined in evaluatingthe response of human macrophages to *Leishmania* infection/interaction. The conclusions from these studies have emphasized the poor/limited responses observed. However, it is clear that at earlier time points after interaction/infection a strong innate immunological response is induced. Further, microarray studies of macrophage infection with other organisms has revealed that there can be a temporal variation/progression with respect to gene induction [30], [83], [84], with an early induction of key immunological genes, that are not evident at later times post-interaction/infection. In this study, a dampening of the response was also noted with time. At 4 hours post-interaction/infection, there is a significant activation of genes involved in cell signaling and the innate immune response yet by 24 hours post-infection, the cells appear to be adapting to parasitism and changes in the metabolic features of the cell become evident. It should be noted that some of the down-regulation of innate immune/inflammatory response-related genes at 24 hours maybe an inevitable result of the *in vitro* system employed for these studies; since the diversity of cell types recruited during infection *in vivo* (and their contributory cytokines/chemokines) that would have further impact on the macrophage response are absent. The induction of both inflammatory and anti-inflammatory pathways is consistent with the mixed cytokine profile found in the acquired immune response to infection with *L. (Viannia)* parasites [6], [8], [12], [13], [81]. Consequently, it is likely that this early response is important in determining the eventual host response to infection and may be representative of the potential acquired immune response that might develop.

Finally, it should be stated that up-regulated gene expression of proteins along any pathway does not necessarily indicate that the function of this pathway is enhanced. Indeed, some transcripts could be increased because of a signal to activate a pathway whereas others could be compensatory and result from a need to modulate the up-regulated expression of an opposing pathway. The microarray data provides a panoramic view of pathways that may be involved in the cellular response to parasite invasion, but the actual nature of this response (as demonstrated for the TLR pathway response [6], [8], [12], [13], [81]) should be further evaluated before conclusions are drawn.

### Comparative analyses with *Leishmania (L.) infantum/chagasi*

Because there are known and pronounced biological differences between the disease presentations resulting from different infecting species of *Leishmania*, comparative analyses were undertaken of MDM gene expression after 16 hours of infection with *L. (L.) infantum/chagasi* (visceral leishmaniasis) or macrophage response 24 hours after infection with *L. (Viannia) panamensis* **(Table S4**). Notably, even at 24 hours post-infection the range of genes whose expression was increased by *L. (V.) panamensis* was higher than those increased for *L. (L.) infantum/chagasi*, although the relative expression of a set of 11 housekeeping genes **(Table S4C**) in infected versus uninfected macrophages (for both species) were comparable. This is significant, as the macrophage response diminishes with time and it would be expected that fewer transcripts would remain elevated in the case of *L. (V.) panamensis*. Indeed, the dominant response to *L. (L.) infantum/chagasi* was down-regulation of gene expression [2]. This predominant down-regulatory response is consistent with other studies comparing the macrophage (mouse and human; different time intervals) responses to other *Leishmania (Leishmania)* organisms [85]. None of the genes whose RNA abundance was found to be up-regulated by macrophages in response to *L. (L.) infantum/chagasi* (9 genes) was found to be significantly up-regulated in response to *L. (Viannia) panamensis*. Of the MDM mRNA levels down regulated by *L. chagasi/infantum* at 16 hours post-infection, most were not found to be significantly changed in response to *L. panamensis* (**Table S4B**).

However, the mRNA levels for two genes, WAS protein family, member 2 (WASF2) and Rap guanine nucleotide exchange factor (RAPGEF1) in contrast to down-regulation after infection with *L. infantum/chagasi*, were up-regulated in response to *L. panamensis* (**Table S4A**). Other mRNA levels unchanged in expression in monocyte-derived macrophages upon *L. infantum/chagasi* infection were also found to be up-regulated upon *L. (Viannia) panamensis* infection. The differentially regulated genes included those that were involved in cell functions related to cell signaling, endocytosis and vesicular trafficking, and cellular adhesion/motility (RASAL2, serine/threonine protein kinase, phospholipase D2, N-myristoyltransferase 2, IPP5, PDLIM5, MYCBP2, PHACTR2) as well as the developing immune response (TRAIL R1, GTPBP, Tec kinase, eotaxin, CD109, IL3R, integrin β3 (CD61), VASP).

The expression of Tec kinase, TRAIL-R1, MYC-binding protein 2, CD109 and eotaxin is consistent with what is known concerning the human inflammatory response to *L. (Viannia)* infection [6], [10], [86]–[88]. Tec kinases are downstream from the activation of TLR [89], [90] receptors and lead to the production of inflammatory cytokines such as TNFα, which is widely associated with pathogenesis of *L. (Viannia)* infection. The ongoing signaling and vesicular trafficking is in contrast to the relatively quiescent state found for human macrophages infected with *L.(L.)infantum/chagasi*. The selective up-regulation of genes modulating the macrophage response indicates a differential response to *L. panamensis* and suggests that the initial innate immune response may lead to and support the development of the non-resolving inflammatory response to infection that is observed clinically.

## Conclusions

Prior studies of macrophage transcriptome indicate that changes in MDM gene expression in response to external stimuli (numbers of genes; inflammatory, anti-inflammatory) are highly variable and dependent upon the particle (inert or pathogen) ingested and nature of the exposure [91]–[94]. After interaction or infection with *L. (V.) panamensis*, results of the current report suggest that the early responses (0 to 4 hours) lead to up-regulation of transcripts contributing to an inflammatory state of the host macrophage. Specifically transcripts encoding proteins involved in cell signaling, inflammation and notably genes involved in the TLR pathways are co-coordinately up-regulated. Simultaneous increased expression of genes associated with anti-inflammatory responses including eicosanoid pathway and transiently IL-10 suggests that a critical balance may be required (between the inflammatory-anti-inflammatory pathways of the macrophage). It is possible that the level of these responses will determine the nature of the acquired immune response that develops and ultimately the outcome of infection or disease. These results substantiate significant differences between the macrophage responses induced by different *Leishmania* species. The macrophage provides critical functions throughout leishmaniasis, including antigen-presentation, parasite containment, and suppression or activation of nearby immune cells through released modulators. Consequently the observed differences in macrophage gene expression may contribute to the host-parasite dynamic in the different forms of leishmaniasis caused by *L. panamensis* and *L. chagasi/infantum*.

## Supporting Information

Table S1 **Experimental conditions and oligonucleotides used for qPCR assays.** Shown are the primers and PCR conditions used for quantitative PCR experiments. All oligonucleotide sets employed were validated using human total RNA (Clontech). IL-1β forward and reverse oligonucleotide primers were based upon those previously described by Goll, et al. [95]. GAPDH oligonucleotide primers were previously described by Carraro, et al. [96]. Remaining oligonucleotides primers were designed using BioEdit (http://www.mbio.ncsu.edu/bioedit/bioedit.html), Primer Express Software (Applied Biosystems), OligoTech (Oligos Etc. Inc. and and Oligo Therapeutics Inc., Wilsonville, OR), and Perl primer (http://perlprimer.sourceforge.net).
(DOC)

Table S2 **Consistently up- and down-regulated genes.** Shown are the level of gene expression, gene bank number and gene description of the annotated genes that were consistently up-regulated and down-regulated at all time points during the first 24 hours of infection (p<0.05, average of the responses of MDM of 6 individuals examined). Genes are organized based upon the relative gene expression at 0.5 hour post-infection/interaction, from highest to lowest.
(DOC)

Table S3 **Pathway analyses of human macrophage response to** ***L. (Viannia) panamensis*** **infection.** Shown are the results from statistical analyses of cellular pathways activated over the first 24 hours of infection. Five pathways were consistently activated throughout the first 24 hours of infection: (1) eicosanoid metabolism, 2) oxidative stress, 3) cadmium induced DNA synthesis and proliferation in macrophages, 4) activation of PKC through G protein coupled receptor and 5) mechanisms of gene regulation by peroxisome proliferators via PPARα). Pathway analysis was performed to identify significantly affected pathways using the pathway database from the Yale Center for Statistical Genomics and Proteomics. The cutoff to cluster similar expressed genes in a significant pathways were 1.5× fold difference between the fluorescence signal intensity from a non-infected control and the *L.(Viannia) panamensis*-infected macrophage with a significance level of ≤0.05. The maximum co-ordinate activation appears at 4 hours; while at 24 hours, pathway analyses indicate ongoing metabolic changes.
(DOC)

Tables S4 **MDM-mRNA level expression post-infection:** ***L. (V.) panamensis*** **–** ***L. (L.) infantum/chagasi*** **.** Shown are the genes whose expression differentially regulated in PBMC-derived macrophages as a consequence of infection. For comparative analyses of the macrophage responses at 24 hours post-infection to *Leishmania (V.) panamensis* versus *L. (L.) chagasi*, the relative microarray fluorescence values from the study of Ettinger and Wilson [2] were used. A) mRNAs Increased in *L. (V.) panamensis* infected MDM and either Unchanged or Reduced expression in *L. (L.) infantum/chagasi* infected MDM; B) mRNA Levels Decreased in MDM infected with *L.(L.) infantum/chagasi* and unchanged in *L(V.) panamensis*; C) Relative mRNA Levels of Housekeeping Genes in MDM infected with *L.(L.) infantum/chagasi* or *L(V.) panamensis*.
(DOC)
