# Supplementary material for: Human Macrophage Response to L. (Viannia) panamensis: Microarray Evidence for an Early Inflammatory Response
Source: PLoS Negl Trop Dis. 2012 Oct 25;6(10):e1866. doi: 10.1371/journal.pntd.0001866 (PMC3493378; doi:10.1371/journal.pntd.0001866)
Supplement: Table S1 — Experimental conditions and oligonucleotides used for qPCR assays. Shown are the primers and PCR conditions used for quantitative PCR experiments. All oligonucleotide sets employed were validated using human total RNA (Clontech). IL-1β forward and reverse oligonucleotide primers were based upon those previously described by Goll, et al. [95]. GAPDH oligonucleotide primers were previously described by Carraro, et al. [96]. Remaining oligonucleotides primers were designed using BioEdit (http://www.mbio.ncsu.edu/bioedit/bioedit.html), Primer Express Software (Applied Biosystems), OligoTech (Oligos Etc. Inc. and and Oligo Therapeutics Inc., Wilsonville, OR), and Perl primer (http://perlprimer.sourceforge.net). (DOC) [file pntd.0001866.s001.doc]

**Table S1:** **Experimental Conditions and Oligonucleotides Used for qPCR Assays**

| **Gene** | **Oligonucleotides Sequence** | **Annealing/Extension Temperature (˚C)**  **and time** |
| --- | --- | --- |
| IL1-β | 5’-TCC TGC GTG TTG AAA GAT GAT-3’  5’-CAA ATC GCT TTT CCA TCT TCT TC-3’ | 63.0 ºC  50 sec |
| TNF-α | 5’-CCC CAG AGG GAA GAG TTC-3’  5’-GGG CTA CAG GCT TGTC ACT-3’ | 62.0 ºC  1 min |
| CSF2(GM) | 5’-CAT GAT GGC CAG CCA CTA C-3’  5’-ATC TGG GTT GCA CAG GAA GT-3’ | 65.0 ºC  1 min |
| PTGS2 | 5’-CCA GAG CAG GCA GAT GAA AT-3’  5’-TGT CAC CAT AGA GTG CTT CCA-3’ | 64.7 ºC  1 min |
| IL6 | 5’-CAG GAG CCC AGC TAT GAA CT-3’  5’-AGC AGC CCC AGG GAG AA-3’ | 64.7 ºC  1 min |
| GAPDH | 5’-TCT GCT CCT CCT GTT CGA C-3’  5’-GAG CGA TGT GGC TCG GCT-3’ | 64.0 ºC  1 min |
| RL18 | 5’-TGG CAC AGG TTG CGA GTT A-3’  5’-ACG AGT GGA GGC CGA AAC-3' | 65.0 ºC  1 min |
