# Supplementary material for: Human Macrophage Response to L. (Viannia) panamensis: Microarray Evidence for an Early Inflammatory Response
Source: PLoS Negl Trop Dis. 2012 Oct 25;6(10):e1866. doi: 10.1371/journal.pntd.0001866 (PMC3493378; doi:10.1371/journal.pntd.0001866)
Supplement: Table S2 — Consistently up- and down-regulated genes. Shown are the level of gene expression, gene bank number and gene description of the annotated genes that were consistently up-regulated and down-regulated at all time points during the first 24 hours of infection (p<0.05, average of the responses of MDM of 6 individuals examined). Genes are organized based upon the relative gene expression at 0.5 hour post-infection/interaction, from highest to lowest. (DOC) [file pntd.0001866.s002.doc]

**Table S2: Consistently* Up- and Down-Regulated Genes**

| **Genbank** | **Description** | **Up-Regulated Fold Change** | | | |
| --- | --- | --- | --- | --- | --- |
|  |  | **0.5 hr** | | **4 hr** | **24 hr** |
| AL031665 | RAD21L1- Novel protein similar to RAD21 homolog, a gene involved in the repair of DNA double-strand breaks | 292.67 | | 222.58 | 79.29 |
| AB065802 | OR52H1; Olfactory receptor family 52, subfamily H, member1 | 259.51 | | 199.08 | 95.61 |
| NM_016429 | COPZ2; Coatomer zeta-2 subunit (Zeta-2 coat protein) | 174.11 | | 132.46 | 75.94 |
| AL020998 | VWA5B1 von Willebrand factor A domain containing 5B1VWAS5B1 | 119.97 | | 65.50 | 39.47 |
| NM_032293 | GARNL3; GTPase activating Rap/ RanGAP domain-like 3 | 93.19 | | 73.45 | 39.63 |
| AK096323 | SGPP2; Sphingosine-1-phosphate phosphatase 2 | 91.06 | | 10.39 | 8.09 |
| AK058062 | FAM81B family with sequence similarity 81, member B | 87.29 | | 105.38 | 29.92 |
| NM_002944 | ROS1; Proto-oncogene tyrosine-protein kinase ROS precursor | 84.58 | | 37.90 | 19.49 |
| NM_004841 | RASAL2; Ras GTPase-activating protein nGAP (RAS protein activator like 2). | 79.41 | | 40.14 | 22.91 |
| AK055765 | ODZ, odd Oz/ten-m homolog 1; belongs to the tenascin family and teneurin subfamily | 77.63 | | 46.16 | 23.62 |
| NM_003215 | TEC; Tyrosine-protein kinase Tec (EC 2.7.1.112). | 73.83 | | 64.94 | 30.97 |
| X76487 | HSEST367 HL 1011 Homo sapiens cDNA clone CHF7 - ribosomal protein L41 (RPL41), transcript variant 2 | 71.56 | | 62.07 | 30.10 |
| NM_002663 | PLD2; Phospholipase D2 (EC 3.1.4.4) (Phosphatidylcholine-hydrolyzing phospholipase D2) | 71.22 | | 73.82 | 23.93 |
| NM_153007 | OPPO 1- protein localized a variety of tissues (spleen, liver, kidney, muscles) | 69.67 | | 59.83 | 48.29 |
| BC000807 | ZNF160; Zinc finger protein 160 (HKr18). | 68.07 | | 39.51 | 22.10 |
| BC014075 | GTPBP1: GTP-binding protein 1 AGP11/GTPBP1 family of GTP-binding proteins | 60.14 | | 63.09 | 29.27 |
| NM_016279 | CDH9; Cadherin-9 precursor. | 54.44 | | 44.91 | 17.59 |
| NM_014588 | VSX1; Visual system homeobox 1 (Transcription factor) (RINX). | 51.59 | | 29.74 | 17.40 |
| AB033104 | STK36; Serine/threonine kinase 36 | 49.08 | | 22.05 | 20.24 |
| AB020691 | RALGAPA1; Ral GTPase activating protein, alpha (catalytic) | 47.57 | | 25.52 | 18.22 |
| X62048 | WEE1; nuclear protein, tyrosine kinase (Ser/Thr family of protein kinases ) | 46.49 | | 30.65 | 12.59 |
| AL161993 | ASPHD2; aspartate beta-hydroxylase domain containing 2 | 39.05 | | 31.50 | 14.37 |
| NM_005085 | NUP214: Nucleoporin; FG-repeat-containing nucleoporins | 32.25 | | 26.73 | 11.26 |
| AF324830 | LILRA5; leukocyte immunoglobulin-like receptor, subfamily A (with TM domain), member 5; induces calcium flux and secretion of proinflammatory cytokines, | 31.61 | | 32.38 | 13.81 |
| BC017943 | PPPR1C; Protein phosphatase 1 regulatory subunit . | 31.53 | | 32.18 | 10.39 |
| AB028962 | RAP1GAP2; RAP1 GTPase activating protein 2 | 23.91 | | 13.37 | 6.92 |
| NM_006990 | WASF2; WAS protein family, member 2; links receptor kinases and actin | 23.09 | | 16.37 | 9.80 |
| BC015993 | FAM151A: family with sequence similarity 151, member A | 22.54 | | 16.28 | 5.73 |
| NM_003844 | TNFRSF10A; Tumor necrosis factor receptor superfamily member 10A precursor (TRAIL-R1). | 22.53 | | 16.17 | 15.55 |
| AY047610 | WFDC9; WAP four-disulfide core domain 9 | 17.94 | | 17.00 | 11.95 |
| NM_006457 | PDLIM5; LIM protein (similar to rat protein kinase C-binding enigma); | 16.79 | | 19.94 | 7.77 |
| D38169 | ITPKC; inositol 1,4,5-trisphosphate 3-kinase C | 16.39 | | 6.94 | 2.24 |
| BC065022 | PCM1; pericentriolar material 1 | 14.40 | | 3.73 | 1.97 |
| AY099296 | Interleukin 27; IL-27 p28 subunit; interleukin 30 | 13.1 | | 3.03 | 2.69 |
| NM_023942 | LRRC61; leucine rich repeat containing 61 | 12.83 | | 9.50 | 6.47 |
| NM_015057 | MYCBP2; MYC binding protein 2; | 12.65 | | 10.56 | 5.83 |
| NM_020371 | AVEN; Cell death regulator Aven; apoptosis, caspase activation inhibitor | 12.25 | | 8.82 | 5.50 |
| NM_001535 | PRMT2; Protein arginine N-methyltransferase 2 (EC 2.1.1.) | 10.70 | | 16.20 | 4.56 |
| AF047002 | THOC4; THO complex 4T; nuclear protein -chaperon | 10.40 | | 2.58 | 1.97 |
| NM_005032 | PLS3; T-plastin. Actin binding | 10.30 | | 7.99 | 5.70 |
| BC012458 | SCFD2; Sec1 family domain containing protein 2 (Syntaxin binding protein 1- like 1) | 8.75 | | 8.27 | 4.58 |
| NM_015179 | RRP12; ribosomal RNA processing 12 homolog (S. cerevisiae) | 8.48 | | 2.39 | 1.76 |
| NM_005427 | TP73; Tumor protein p73 (p53-like transcription factor) | 8.47 | | 2.16 | 2.31 |
| NM_001915 | CYB561; Cytochrome b561 (Cytochrome b-561) | 7.51 | | 2.15 | 1.69 |
| Y08890 | IPO5; importin 5 Importin alpha binds the NLS-containing cargo in the cytoplasm | 6.37 | | 4.18 | 3.02 |
| NM_005614 | RHEB; GTP-binding protein Rheb | 4.22 | | 2.98 | 2.26 |
| NM_153813 | ZFPM1; Zinc finger protein multitype 1 | 4.22 | | 2.13 | 1.71 |
| AK092906 | EXOC3L1; Exocyst complex component 3-like | 4.16 | | 2.10 | 1.78 |
| AB033049 | ANKRD50; Ankyrin repeat domain 50 | 4.15 | | 2.95 | 1.75 |
| NM_005738 | ARLA4; ADP-ribosylation factor-like protein 4A ; GTP-binding protein; nuclear and extra-nuclear | 3.55 | | 3.93 | 2.42 |
| NM_004233 | CD83; CD83 molecule (HB15) | 2.33 | | 3.07 | 1.64 |
| NM_002228 | JUN; Jun proto-oncogene | 2.31 | | 2.13 | 1.67 |
| BC010123 | SH3BP5; SH3 domain-binding protein 5 (preferentially associates with BTK). | 2.04 | | 3.12 | 2.13 |
| NM_000362 | TIMP-3; Tissue inhibitor of metalloproteinases-3 (involved in degradation of the extracellular matrix) | 1.99 | | 2.25 | 1.88 |
| NM_005239 | ETS2; v-ets erythroblastosis virus E26 oncogene homolog 2; transcription factor | 1.7 | | 7.59 | 4.30 |
|  |  |  | |  |  |
| **Genbank** | **Description** | **Down-Regulated Fold Change** | | | |
|  |  | **0.5 hr** | **4 hr** | | **24 hr** |
| NM_002713 | PPP1R8, Protein phosphatase 1, regulatory (inhibitor) subunit 8 transcript | -7.90 | -11.58 | | -7.92 |
| NM_000053 | ATP7B; Copper-transporting ATPase 2 (EC 3.6.3.4) (Copper pump 2) | -8.46 | -14.91 | | -10.15 |
| NM_080805 | COL13A1 ; Collagen, type XIII, alpha 1, transcript variant 9 | -8.57 | -23.07 | | -60.28 |
| NM_014055 | IFT81; Intraflagellar transport 81 homolog (*Chlamydomonas*) transcript variant 1 | -12.05 | -22.99 | | -17.81 |
| NM_001123041 | CCR2; Chemokine (C-C motif) receptor 2 (MCP-1 Receptor) | -11.82 | -21.28 | | -28.62 |
| NM_032037 | TSSK6; Serine/threonine protein kinase | -14.55 | -19.79 | | -14.29 |
| AJ301580 | **DMRTA2;**  DMRT-like family A2 | -16.15 | -28.87 | | -51.00 |
| AK022308 | cDNA FLJ12246 fis, clone MAMMA1001343 | -17.53 | -32.91 | | -74.78 |
| AK074414 | CDHR3; cadherin-related family member 3 | -17.73 | -37.51 | | -68.03 |
| NM_002270 | TNPO1; Transportin 1; (beta subunit of the karyopherin receptor) | -19.99 | -15.34 | | -12.53 |
| NM_013355 | PKN3; Protein kinase N3 | -21.05 | -22.08 | | -25.27 |
| BC009393 | AGFG2; ArfGAP with FG repeats 2; HIV-1 Rev binding protein-like protein (RAB-R); mediates nucleocytoplasmic transfer | -23.56 | -52.89 | | -24.48 |
| NM_002861 | PCYT2 ; Phosphate cytidylyltransferase 2, ethanolamine | -25.69 | -63.59 | | -25.40 |
| AK025174 | cDNA: FLJ21521 fis, clone COL05880- Gasdermin-like (GSDML) | -25.80 | -35.76 | | -35.99 |
| AK057477 | HYLS1- hydrolethalus syndrome 1 | -26.07 | -15.98 | | -14.69 |
| BC007657 | Ubiquitin-conjugating enzyme E2 M (EC 6.3.2.19) (Ubiquitin-protein ligase M) (Ubiquitin carrier protein M) (Nedd8-conjugating enzyme Ubc12). | -26.94 | -40.40 | | -20.18 |
| AY358948 | SPON2; Spondin 2, extracellular matrix protein | -27.17 | -49.84 | | -66.23 |
| AC005175 | TBXA2R; thromboxane A2 receptor | -37.05 | -55.53 | | -38.60 |
| AK056288 | ZNF407; Zinc finger protein 407 | -41.19 | -32.80 | | -58.68 |
| BC030711 | APFL aprataxin and PNKP like factor | -41.74 | -52.80 | | -62.16 |
| AB067479 | DDB1 and CUL4 associated factor 12 WD repeat-containing protein that interacts with the COP9 signalosome | -41.78 | -27.89 | | -11.70 |
| NM_025048 | G protein-coupled receptor 110 (GPR110) | -42.18 | -75.27 | | -28.62 |
| NM_002246 | Potassium channel, subfamily K, member 3 (KCNK3) | -42.37 | -85.43 | | -76.59 |
| NM_014799 | Hephaestin isoform b | -45.32 | -92.62 | | -97.16 |
| NM_005298 | G protein-coupled receptor GPR25. | -45.65 | -89.79 | | -100.00 |
| AK098687 | Probable ribonuclease C14orf6 precursor (EC 3.1.27.-) | -78.96 | -69.16 | | -75.21 |
|  | | | | | |

* Up- or Down-regulated at all 3 time points (0.5, 4.0 and 24 hours)
