# Supplementary material for: Human Macrophage Response to L. (Viannia) panamensis: Microarray Evidence for an Early Inflammatory Response
Source: PLoS Negl Trop Dis. 2012 Oct 25;6(10):e1866. doi: 10.1371/journal.pntd.0001866 (PMC3493378; doi:10.1371/journal.pntd.0001866)
Supplement: Table S3 — Pathway analyses of human macrophage response to L. (Viannia) panamensis infection. Shown are the results from statistical analyses of cellular pathways activated over the first 24 hours of infection. Five pathways were consistently activated throughout the first 24 hours of infection: (1) eicosanoid metabolism, 2) oxidative stress, 3) cadmium induced DNA synthesis and proliferation in macrophages, 4) activation of PKC through G protein coupled receptor and 5) mechanisms of gene regulation by peroxisome proliferators via PPARα). Pathway analysis was performed to identify significantly affected pathways using the pathway database from the Yale Center for Statistical Genomics and Proteomics. The cutoff to cluster similar expressed genes in a significant pathways were 1.5× fold difference between the fluorescence signal intensity from a non-infected control and the L.(Viannia) panamensis-infected macrophage with a significance level of ≤0.05. The maximum co-ordinate activation appears at 4 hours; while at 24 hours, pathway analyses indicate ongoing metabolic changes. (DOC) [file pntd.0001866.s003.doc]

**TABLE S3: Pathway Analyses of Human Macrophage Response to *L. (Viannia) panamensis* Infection**

| **Coordinately Regulated Gene Pathways** | | | | | | | | |
| --- | --- | --- | --- | --- | --- | --- | --- | --- |
| **0.5 HOURS** | | **P-Value** | | **4 HOURS** | **P-Value** | **24 HOURS** | | **P-Value** |
| Cadmium induces DNA synthesis and proliferation in macrophages(BC) | | 0.000822 | | Cadmium induces DNA synthesis and proliferation in macrophages(BC) | 0.000117 | Cadmium induces DNA synthesis and proliferation in macrophages(BC) | | 0.005695 |
| Eicosanoid Metabolism(BC) | | 0.002895 | | Eicosanoid Metabolism(BC) | 0.033535 | Eicosanoid Metabolism(BC) | | 0.003249 |
| Activation of PKC through G protein coupled receptor(BC) | | 0.014759 | | Activation of PKC through G protein coupled receptor(BC) | 0.020006 | Activation of PKC through G protein coupled receptor(BC) | | 0.012126 |
| Mechanism of Gene Regulation by Peroxisome Proliferators via PPARα | | 0.019402 | | Mechanism of Gene Regulation by Peroxisome Proliferators via PPARα (BC) | 0.000075 | Mechanism of Gene Regulation by Peroxisome Proliferators via PPARα (BC) | | 0.000125 |
| Oxidative Stress(GenMapp) | | 0.045693 | | Oxidative Stress(GenMapp) | 0.000239 | Oxidative Stress (GenMapp) | | 0.000085 |
|  | | | | | | | | |
| The information-processing pathway at the IFN-beta enhancer(BC) | | 0.007567 | | The information-processing pathway at the IFN-beta enhancer(BC) | 0.000863 |  | |  |
| Pertussis toxin-insensitive CCR5 Signaling in Macrophage(BC) | | 0.010854 | | Pertussis toxin-insensitive CCR5 Signaling in Macrophage(BC) | 0.014081 |  | |  |
| METS affect on Macrophage Differentiation(BC) | | 0.001638 | | METS affect on Macrophage Differentiation(BC) | 0.002379 |  | |  |
| Oxidative Stress Induced Gene Expression Via Nrf2(BC) | | 0.002421 | | Oxidative Stress Induced Gene Expression Via Nrf2(BC) | 0.003578 |  | |  |
| NFkB activation by Nontypeable Hemophilus influenzae(BC) | | 0.028004 | | NFkB activation by Nontypeable Hemophilus influenzae(BC) | 0.000001 |  | |  |
| ATM Signaling Pathway(BC) | | 0.017112 | | ATM Signaling Pathway(BC) | 0.000002 |  | |  |
| Apoptosis Mechanisms(GenMapp) | | 0.022552 | | Apoptosis Mechanisms(GenMapp) | 0.000265 |  | |  |
| Regulation of MAP Kinase Pathways Through Dual Specificity Phosphatases(BC) | | 0.024388 | | Regulation of MAP Kinase Pathways Through Dual Specificity Phosphatases(BC) | 0.00349 |  | |  |
| IL 6 signaling pathway (BC) | | 0.025006 | | IL 6 signaling pathway(BC) | 0.033535 |  | |  |
| Toll-like receptor signaling pathway (BC) | | 0.025156 | | Toll-Like Receptor Pathway(BC) | 0.031605 |  | |  |
| Adipogenesis Human(GenMapp) | | 0.044783 | | Adipogenesis Human(GenMapp) | 0.005242 |  | |  |
|  | |  | | | | | | |
|  | |  | | Prostaglandin Synthesis & Regulation (GenMapp) | 0.0462 | Prostaglandin Synthesis & Regulation (GenMapp) | | 0.038563 |
|  | |  | | Matrix Metalloproteinases(GenMapp) | 0.00305 | Matrix Metalloproteinases(GenMapp) | | 0.038318 |
|  | |  | | Inhibition of Matrix Metalloproteinases (BC) | 0.032848 | Inhibition of Matrix Metalloproteinases(BC) | | 0.001852 |
|  | |  | | Genes specific to blood and lymph tissue 1 (GenMapp) | 0.012414 | Genes specific to blood and lymph tissue 2 (GenMapp) | | 0.005188 |
| **Selectively Expressed Pathways** | | | | | | | | |
| Role of b-arrestins in the activation and targeting of MAP kinases(BC) | 0.010854 | | Toll-like receptor signaling pathway | | <0.000001 | | Glutathione metabolism | 0.000125 |
| Small Ligand GPCRs(GenMapp) | 0.014846 | | Cytokines and Inflammatory Response (BioCarta)(GenMapp) | | 0.000001 | | D-Glutamine and D-glutamate metabolism | 0.001852 |
| Signaling Pathway from G-Protein Families(BC) | 0.017187 | | MAPK signaling pathway | | 0.000004 | | Phospholipids as signalling intermediaries(BC) | 0.003249 |
| Activation of cAMP-dependent protein kinase, PKA(BC) | 0.01932 | | TNFR2 Signaling Pathway(BC) | | 0.000016 | | Generation of amyloid b-peptide by PS1(BC) | 0.004513 |
| Roles of ß-arrestin-dependent Recruitment of Src Kinases in GPCR Signaling(BC) | 0.01956 | | Signal transduction through IL1R(BC) | | 0.000001 | | Proteasome | 0.006379 |
| Nuclear Receptors(GenMapp) | 0.02446 | | TNFR2 Signaling Pathway(BC) | | 0.000016 | | Integrin-mediated cell adhesion(GenMapp) | 0.008806 |
| Phospholipase C-epsilon pathway(BC) | 0.00618 | | IL-10 Anti-inflammatory Signaling Pathway(BC) | | 0.000083 | | Pentose Phosphate Pathway(GenMapp) | 0.008806 |
|  |  | | TNF/Stress Related Signaling(BC) | | 0.000154 | | Phospholipase C d1 in phospholipid associated cell signaling(BC) | 0.011187 |
|  |  | | Cells and Molecules involved in local acute inflammatory response(BC) | | 0.000218 | | Proteasome Degradation(GenMapp) | 0.011187 |
|  |  | | MAPKinase Signaling Pathway(BC) | | 0.000436 | | Nitrogen metabolism | 0.016934 |
|  |  | | Cytokine Network(BC) | | 0.000921 | | Selenoamino acid metabolism | 0.029345 |
|  |  | | NF-kB Signaling Pathway(BC) | | 0.000921 | | Glyoxylate and dicarboxylate metabolism | 0.035086 |
|  |  | | AKT Signaling Pathway(BC) | | 0.002937 | |  |  |
|  |  | | Chaperones modulate interferon Signaling Pathway(BC) | | 0.002937 | |  |  |
|  |  | | Acetylation and Deacetylation of RelA in The Nucleus(BC) | | 0.016692 | |  |  |
|  |  | | Msp/Ron Receptor Signaling Pathway(BC) | | 0.001095 | |  |  |
|  |  | | CD40L Signaling Pathway(BC) | | 0.001147 | |  |  |
|  |  | | Adhesion and Diapedesis of Granulocytes(BC) | | 0.00149 | |  |  |
|  |  | | Regulation of hematopoiesis by cytokines(BC) | | 0.00149 | |  |  |
|  |  | | The 4-1BB-dependent immune response(BC) | | 0.002379 | |  |  |
