# Supplementary material for: Human Macrophage Response to L. (Viannia) panamensis: Microarray Evidence for an Early Inflammatory Response
Source: PLoS Negl Trop Dis. 2012 Oct 25;6(10):e1866. doi: 10.1371/journal.pntd.0001866 (PMC3493378; doi:10.1371/journal.pntd.0001866)
Supplement: Tables S4 — MDM-mRNA level expression post-infection: L. (V.) panamensis – L. (L.) infantum/chagasi . Shown are the genes whose expression differentially regulated in PBMC-derived macrophages as a consequence of infection. For comparative analyses of the macrophage responses at 24 hours post-infection to Leishmania (V.) panamensis versus L. (L.) chagasi, the relative microarray fluorescence values from the study of Ettinger and Wilson [2] were used. A) mRNAs Increased in L. (V.) panamensis infected MDM and either Unchanged or Reduced expression in L. (L.) infantum/chagasi infected MDM; B) mRNA Levels Decreased in MDM infected with L.(L.) infantum/chagasi and unchanged in L(V.) panamensis; C) Relative mRNA Levels of Housekeeping Genes in MDM infected with L.(L.) infantum/chagasi or L(V.) panamensis. (DOC) [file pntd.0001866.s004.doc]

**Tables S4:** MDM-mRNA Level Expression Post-infection: *L. (V.) panamensis* – *L. (L.) infantum/chagasi*

**S4A. mRNAs Increased in *L. (V.) panamensis* infected MDM and either Unchanged or Reduced expression in *L. (L.) infantum/chagasi*  infected MDM**

| **Common Name/ Symbol** | **GenBank Accession Number** | **Gene Description** |
| --- | --- | --- |
| COPZ2 | NM_016429 | **Nonclathrin coat protein zeta-COP** |
| ODF4 | NM_153007 | **OPPO 1** This gene encodes a protein that is localized a variety of tissues (spleen, liver, kidney, muscles) |
| GARNL3 | NM_032293 | **GTPase activating Rap/RanGAP** domain-like 3 |
| VWA5B1 | AL020998 | **von Willebrand factor A domain containing 5B1** |
| TEC | NM_003215 | **Tec protein tyrosine kinase:** Tec regulates M-CSFR signaling-induced macrophage survival and cooperates with Jak2 kinases to mediate cytokine-driven activation of cfos transcription |
| FAM81B | AK058062 | **FAM81B family** with sequence similarity 81, member B |
| GTPBP1 | BC014075 | **GTP binding protein 1:** This protein is a member of the AGP11/GTPBP1 family of GTP-binding proteins and is upregulated by interferon-gamma |
| PLD2 | NM_002663 | **Phospholipase D2**; Involved in chemotaxis and cell signaling |
| RASAL2 | NM_004841 | **Ras GTPase-activating protein nGAP** is an enzyme that is encoded by the *RASAL2* gene. GAPs function as activators of Ras superfamily of small GTPases. |
| STK36 | AB033104 | **Serine/threonine kinase 36** |
| ROS1 | NM_002944 | **V-ros UR2 sarcoma virus oncogene homolog 1;** The protein encoded by this gene is a type I integral membrane protein with tyrosine kinase activity. |
| VSX1 | NM_014588 | **Visual system homeobox 1 homolog** |
| TNFRSF10A | NM_003844 | **TRAIL R1-Tumor necrosis factor receptor superfamily, member 10**; TNFRs trigger multiple signal transduction pathways involved in inflammation, and apoptosis. |
| ASPHD2 | AL161993 | **Aspartate beta-hydroxylase** domain containing 2 (ASPH)  Aspartyl (asparaginyl) b-hydroxylase hydroxylates aspartate and asparagine residues in EGF-like domains of proteins. |
| PPP1R1C; IPP5 | BC017943 | **Protein phosphatase 1, regulatory (inhibitor) subunit 1C (IPP5);** Protein phosphatase-1 (PP1) is a major serine/threonine phosphatase that regulates a variety of cellular functions (cell cycle, apoptosis, respiratory burst). |
| WASF2 | NM_006990 | **WAS protein family, member 2 IMD2**; The gene product is a protein that forms a multiprotein complex that links receptor kinases and actin |
| PDLIM5 | NM_006457 | **LIM protein** (similar to rat protein kinase C-binding enigma)  The LIM domains bind highly diverse partners, ranging from signaling molecules, actin cytoskeletal components to transcription factors, which support cellular functions including, actin organization, integrin-dependent adhesion and signaling, and cell-fate determination. |
| LRRC61 | NM_023942 | **Leucine rich repeat containing 61**; Protein-protein interactions |
| MYCBP2 | NM_015057 | **MYC binding protein 2**; E3 ubiquitin-protein ligase MYCBP2; inhibits p38 MAPK signaling by targeting MAPKKK12; also effects Myc and mTOR signaling (type INFs and IL-10). |
| CCL24/  Eotaxin2 | NM_002991 | **Small inducible cytokine subfamily A (Cys-Cys), member 24;** eotaxin2 involved in inflammation recruitment of eosinophils and macrophages |
| CD109 | AF410459 | **CD109**; **Member of alpha2-macroglobulin/complement superfamily**; a GPI-linked glycoprotein that can bind to and negatively regulate signaling of transforming growth factor beta. |
| CRIPT | NM_014171 | **Cysteine-rich PDZ-binding protein**; Microtubule-associated protein CRIPT |
| ITGB3 | NM_000212 | **Integrin, beta 3** (platelet glycoprotein IIIa, antigen CD61) involved in recognition and internalization of apoptotic cells. |
| IL3RA | NM_002183 | **Interleukin 3 receptor**, alpha (low affinity) |
| ZNF267 | NM_003414 | **Zinc finger protein 267;** a proto-oncogenic transcription factor. Plays a role in cell proliferation; apoptosis |
| NMT2 | NM_004808 | **N-myristoyltransferase 2**; N-myristoyltransferase (NMT) catalyzes the reaction of N-terminal myristoylation of many signaling proteins |
| CNIH3 | AF070524 | **Cornichon homolog 3**; signaling; integral membrane |
| CD98HC; SLC3A2 | NM_002394 | **Solute carrier family 3 (activators of dibasic and neutral amino acid transport), member 2** |
| MGST1 | BC005923 | **Microsomal glutathione S-transferase 1** (EC 2.5.1.18) (Microsomal GST- 1) |
| MFSD11 | NM_024311 | **Major facilitator superfamily domain containing 11** |
| PSMB7 | NM_002799 | **Proteasome (prosome, macropain) subunit, beta type, 7**; a member of the proteasome B-type family( T1B family) which is downregulated by gamma interferon |
| TP73 | NM_005427 | **Tumor protein p73**; a member of the p53 family of transcription factors involved in cellular responses to stress and development. |
| PHACTR2 | NM_014721 | **Phosphatase and actin regulator 2**; phosphatase inhibitor; actin binding |
| PKM2 | NM_002654 | **Pyruvate kinase**; Catalyzes the transfer of a phosphoryl group from phosphoenolpyruvate to ADP, generating ATP and pyruvate. |
| SLC44A1 | AJ420812 | **Solute carrier family 44,** member 1; amino acid/choline transmembrane transporter activity; CDw92 antigen |
| RAI14 | NM_015577 | **Retinoic acid induced 14;** ankyrin repeat and coiled-coil structure-containing protein |
| RDX | NM_002906 | **Radixin**; A cytoskeletal protein that may be important in linking actin to the plasma membrane. |
| HIST2H2AA3 | NM_003516 | **Histone cluster 2, H2aa3** (HIST2H2AA3), |
| RAPGEF1 | NM_005312 | **Rap guanine nucleotide exchange factor** (GEF) 1 |
| NRIP3 | NM_020645 | **Nuclear receptor interacting protein 3** (Sarcoma antigen NY-SAR-105). aspartic endopeptidase activity |
| ABHD2 | NM_007011 | **Abhydrolase domain containing protein 2** (Protein PHPS1-2) |
| EPHB3 | NM_004443 | **EPH receptor B3**; Ephrin-B (EFNB) class receptors are transmembrane proteins; Ephrin receptors make up the largest subgroup of the receptor tyrosine kinase (RTK) family. |
| TGM2 | NM_004613 | **Transglutaminase 2** (C polypeptide, protein-glutamine-gamma-glutamyltransferase) |
| NCBP1 | NM_002486 | **Nuclear cap binding protein subunit 1**; Component of the nuclear cap-binding protein complex (CBC). |
| NEDD1 | BC027605 | **Neural precursor cell expressed, developmentally down-regulated 1**; interaction with gamma-tubulin for targeting the gammaTuRC to the centrosome; spindle formation |
| VASP | NM_003370 | **Vasodilator-stimulated phosphoprotein (VASP**); associated with filamentous actin formation; role in cell adhesion, motility and intracellular signaling pathways that regulate integrin-extracellular matrix interactions. |
| CSF2RA | NM_006140 | **Colony stimulating factor 2 receptor**, alpha, low-affinity (granulocyte-macrophage) |

**S4B. mRNA Levels Decreased in MDM infected with *L.(L.) infantum/chagasi* and unchanged in *L(V.) panamensis***

| **Common Name/ Symbol** | **GenBank Accession Number** | **Gene Description** |
| --- | --- | --- |
| ORC4L | NM_002552 | **Origin recognition complex**, subunit 4-like (yeast) |
| FAM20A | AK056789 | **Family with sequence similarity 20**, member A |
| COL23A1 | AL137461 | **Collagen, type XXIII,** alpha 1 |
| CLN8 | NM_018941 | **Ceroid-lipofuscinosis**, neuronal 8 (epilepsy, progressive with mental retardation) |
| ANKRD10 | NM_017664 | **Ankyrin repeat domain** 10 |
| IL1R2 | NM_004633 | **Interleukin 1 receptor**, type II |
| TNFSF15 | NM_005118 | **Tumor necrosis factor (ligand)** superfamily, member 15 |
| TPR | NM_003292 | **Translocated promoter region** (to activated MET oncogene) |
| ACSL4 | NM_022977 | **Acyl-CoA synthetase** long-chain family member 4 |
| PHGDH | BC011262 | **Phosphoglycerate dehydrogenase** |
| YAF2 | NM_005748 | **YY1 associated factor 2** |
| SCML1 | NM_006746 | **Sex comb on midleg-like 1** (Drosophila) |
| PCTK2 | NM_002595 | **PCTAIRE protein kinase** **2** |
| SIGLEC10 | NM_033130 | **Sialic acid binding Ig-like lectin** 10 |
| FMNL3 | AL162062 | **Formin-like 3** |
| PAK1 | NM_002576 | **p21/Cdc42/Rac1-activated kinase 1** (STE20 homolog, yeast) |
| SLC16A6 | NM_004694 | **Solute carrier family** 16 (monocarboxylic acid transporters), member 6 |
| ARF1 | NM_001658 | **ADP-ribosylation factor 1** |
| MLL | L04731 | **Myeloid/lymphoid or mixed-lineage leukemia** (trithorax homolog, Drosophila) |
| PRDX6 | NM_004905 | **Peroxiredoxin 6** |
| PPP1R13B | AJ318887 | **Protein phosphatase 1, regulatory (inhibitor)** subunit 13B |
| ZNF286 | AB058777 | **Zinc finger protein 286** |
| TNFRSF10B | AF016266 | **Tumor necrosis factor receptor superfamily**, member 10b |
| DPEP2 | NM_022355 | **Dipeptidase 2** |
| MLL3 | NM_001187 | **Myeloid/lymphoid or mixed-lineage leukemia 3** |
| IL1R2 | NM_004633 | **Interleukin 1 receptor**, type II |
| EIF2C3 | NM_024852 | **Eukaryotic translation initiation factor** 2C, 3 |
| SLC16A7 | NM_004731 | **Solute carrier family 16** (monocarboxylic acid transporters), member 7 |
| NDUFB8 | NM_005004 | **NADH dehydrogenase** (ubiquinone) 1 beta subcomplex, 8, 19kDa |
| MAK3 | NM_025146 | **Mak3 h**omolog (S. cerevisiae) |
| DOCK5 | NM_024940 | **Dedicator of cytokinesis** 5 |
| SENP6 | NM_015571 | **SUMO1/sentrin specific peptidase** 6 |
| TPM2 | NM_003289 | **Tropomyosin 2** (beta) |
| RNF125 | NM_017831 | **Ring finger protein** 125 |
| GDF15 | NM_004864 | **Growth differentiation factor** 15 |
| FALZ | AB032251 | **Fetal Alzheimer antigen** |
| GDAP2 | NM_017686 | **Ganglioside induced differentiation associated protein** 2 |
| MGA | AB011090 | **MAX gene associated** |
| ZDHHC4 | NM_018106 | **Zinc finger**, DHHC-type containing 4 |
| ACE | NM_000789 | **Angiotensin I converting enzyme** (peptidyl-dipeptidase A) 1 |
| NKTR | NM_005385 | **Natural killer-tumor recognition** sequence |

**S4C. Relative mRNA Levels of Housekeeping Genes in MDM infected with *L.(L.) infantum/chagasi* or *L(V.) panamensis***

| **GENE Designation** | **GenBank Accession Number** | **Gene Symbol** | **Ratio: Infected/ Non-infected**  ***L. (L.) infantum/ chagasi*** | **Ratio: Infected/ Non-infected**  ***L. (V.) panamensis*** |
| --- | --- | --- | --- | --- |
| Glyceraldehyde-3-phosphate dehydrogenase | [NM_001256799](http://www.ncbi.nlm.nih.gov/nuccore/NM_001256799.1) | GAPDH | 1.045260459 | 0.85926336 |
| Actin, beta | [NM_001101](http://www.ncbi.nlm.nih.gov/nuccore/NM_001101.3) | ACTB | 0.985656655 | 0.7919765 |
| Ribosomal protein S3 | NM_001005 | RPS3 | 0.79085329 | 0.796559 |
| Non-POU-domain-containing, octamer-binding | NM_007363 | NONO | 0.842464844 | 0.84282017 |
| Ribosomal protein S29 | NM_001032 | RPS29 | 1.008539691 | 0.8668699 |
| Ribosomal protein L19 | NM_000981 | RPL19 | 0.705089007 | 0.7807884 |
| Heat shock 90kD protein 1, alpha | AK056446 | HSPCA | 0.946933737 | 1.1056 |
| Ribosomal protein L11 | NM_000975 | RPL11 | 0.761265058 | 0.6664284 |
| Tubulin, alpha 1 (testis specific) | AK054731 | TUBA1 | 0.986426639 | 0.9583955 |
| Hypoxanthine phosphoribosyltransferase 1 | NM_000194 | HPRT1 | 0.942197668 | 1.0414269 |
| Vimentin | NM_003380 | VIM | 1.018805597 | 1.1311055 |
